# Genetic Loci Associated with Alzheimer’s Disease and Cerebrospinal Fluid Biomarkers in a Finnish Case-Control Cohort

**DOI:** 10.1371/journal.pone.0059676

**Published:** 2013-04-03

**Authors:** Lyzel S. Elias-Sonnenschein, Seppo Helisalmi, Teemu Natunen, Anette Hall, Teemu Paajanen, Sanna-Kaisa Herukka, Marjo Laitinen, Anne M. Remes, Anne M. Koivisto, Kari M. Mattila, Terho Lehtimäki, Frans R. J. Verhey, Pieter Jelle Visser, Hilkka Soininen, Mikko Hiltunen

**Affiliations:** 1 Department of Psychiatry and Neuropsychology, School for Mental Health and Neuroscience, Alzheimer Center Limburg, Maastricht University, Maastricht, The Netherlands; 2 Institute of Clinical Medicine-Neurology, University of Eastern Finland and Department of Neurology, Kuopio University Hospital, Kuopio, Finland; 3 Department of Clinical Chemistry, Fimlab Laboratories and School of Medicine, University of Tampere, Tampere, Finland; 4 Department of Neurology, Alzheimer Center, VU University Medical Center Amsterdam, Amsterdam, The Netherlands; UCL Institute of Neurology, United Kingdom

## Abstract

**Objectives:**

To understand the relation between risk genes for Alzheimer’s disease (AD) and their influence on biomarkers for AD, we examined the association of AD in the Finnish cohort with single nucleotide polymorphisms (SNPs) from top AlzGene loci, genome-wide association studies (GWAS), and candidate gene studies; and tested the correlation between these SNPs and AD markers Aβ_1–42_, total tau (t-tau), and phosphorylated tau (p-tau) in cerebrospinal fluid (CSF).

**Methods:**

We tested 25 SNPs for genetic association with clinical AD in our cohort comprised of 890 AD patients and 701-age matched healthy controls using logistic regression. For the correlational study with biomarkers, we tested 36 SNPs in a subset of 222 AD patients with available CSF using mixed models. Statistical analyses were adjusted for age, gender and *APOE* status. False discovery rate for multiple testing was applied. All participants were from academic hospital and research institutions in Finland.

**Results:**

*APOE*-ε4, *CLU* rs11136000, and *MS4A4A* rs2304933 correlated with significantly decreased Aβ_1–42_ (corrected p<0.05). At an uncorrected p<0.05, *PPP3R1* rs1868402 and *MAPT* rs2435211 were related with increased t-tau; while *SORL1* rs73595277 and *MAPT* rs16940758, with increased p-tau. Only *TOMM40* rs2075650 showed association with clinical AD after adjusting for *APOE*-ε4 (p = 0.007), but not after multiple test correction (p>0.05).

**Conclusions:**

We provide evidence that *APOE*-ε4, *CLU* and *MS4A4A,* which have been identified in GWAS to be associated with AD, also significantly reduced CSF Aβ_1–42_ in AD. None of the other AlzGene and GWAS loci showed significant effects on CSF tau. The effects of other SNPs on CSF biomarkers and clinical AD diagnosis did not reach statistical significance. Our findings suggest that *APOE*-ε4, *CLU* and *MS4A4A* influence both AD risk and CSF Aβ_1–42_.

## Introduction

Alzheimer’s disease (AD) is a neurodegenerative disease with a complex etiology. Neuritic plaques mainly composed of aggregated β-amyloid (Aβ) and neurofibrillary tangles (NFTs) resulting from hyperphosporylated tau protein (p-tau) are pathological hallmarks of AD [1]. Aβ_1–42_ tends to aggregate more compared to other Aβ isoforms [2]. Total tau (t-tau) concentrations in cerebrospinal fluid (CSF) have been suggested to indicate the extent of neuronal damage, while p-tau levels reflected the phosphorylated state of tau [3]. P-tau and t-tau levels signal axonal degeneration [4], [5]. In AD, the concentration of CSF Aβ_1–42_ is decreased, which is supposed to reflect sequestration of Aβ_1–42_ in amyloid plaques in the brain [6], while t-tau and p-tau levels are increased [1].

The majority of AD cases have been reported to have a strong genetic component [7]. The apolipoprotein E (*APOE*) ε4 allele is the strongest known genetic risk factor for AD. Other high-risk genetic variants have been identified in genome-wide association studies (GWAS) (for review, see [8]). In addition, previous studies showed that a number of candidate genes correlated with Aβ or tau. However, the relation between these AD risk genes and AD biomarkers remains ambiguous. With the exception of *APOE* and translocase of outer mitochondrial membrane 40 homolog (*TOMM40*), single nucleotide polymorphisms (SNPs) identified in case-control GWAS with clinical AD as outcome have not been replicated in GWAS with biomarkers as outcome [9].

In the study, we first investigated whether previously reported genetic risk factors for AD were associated with AD risk in a Finnish case-control cohort. Second, we tested in the AD group the effects of these variants on the AD markers Aβ_1–42_ and tau in CSF. We selected SNPs from AlzGene and GWAS, and from candidate genes that previously showed a relation with CSF Aβ_1–42_ and tau.

## Materials and Methods

### Study Population

The Finnish-AD is a multicenter cohort comprised of 890 AD patients and 701 age-matched healthy controls from Kuopio, Oulu, and Tampere in Finland. All patients were diagnosed with probable AD according to the National Institute of Neurological and Communicative Disorders and Stroke and the Alzheimer’s Disease and Related Disorders Association (NINCDS-ADRDA) criteria [10]. AD patients with an early onset did not show conclusive evidence of autosomal dominant transmission or mutations in the amyloid precursor protein (*APP*), presenilin 1 (*PSEN1*), or presenilin 2 (*PSEN2*) genes. Control subjects had no symptoms of cognitive impairment based on clinical interview and neuropsychological examination. Of the 890 AD patients, 222 from Kuopio provided CSF for our study.

The Ethics Committee of the North-Savo Hospital District, Kuopio Finland, approved the study. The physician and/or the study nurse gave written information of the study and explained the study protocol to the patient and caretaker if available. All participants provided written informed consent. A next of kin, caretakers or guardians consented on the behalf of participants whose capacity to consent was compromised. In these cases also the patient's own opinion was asked and considered, and the patient was recruited in the study only when he or she also agreed with this. The ethics committee approved this informed consent procedure.

### Gene Selection

We selected genes based on their reported association with AD or effect on CSF Aβ and tau. We included the 10 ‘top results’ loci from AlzGene, which is an online database providing meta-analyses of published genetic association studies (for AlzGene top results criteria, see [11]), and selected the most promising SNPs from GWAS or other candidate gene studies. *APOE, CR1, BIN1, CD2AP, CLU, MS4A4E, MS4A6A, PICALM, ABCA7,* and *CD33* were from AlzGene (for gene names, see Table S1). Analyses with *APOE* alone were performed for reference purposes, as *APOE* is an established susceptibility gene for sporadic AD [11]–[13]. Functions of the AlzGene variants in relation to AD have been previously described [9], [14], [15]. *MS4A4A*, *EXOC3L2* and *MTHFD1L* have been shown in GWAS to be associated with AD [16]–[18] but have not been included in the AlzGene top list to date.

*CYP19A* and *TOMM40* have been shown in GWAS with biomarkers as outcome to be related to Aβ, whereas *EPC2* and *RELN* were associated with tau [19]–[21]. *CYP19A* has also been reported to increase AD risk [22].

The candidate genes we selected that have been studied in relation to CSF biomarkers were *ACE, IDE*, *MAPT, SORL1, CYP46A1, BDNF, TF*, *PPP3R1,* and another *TOMM40* polymorphism. The effects of *ACE*, *MAPT*, *SORL,* and *TOMM40* on Aβ and tau have been reported in a review (see [9]). *PPP3R1* has been correlated with increased p-tau [4]. *BDNF* was linked with decreased total Aβ while *TF* was related to decreased Aβ_1–42_/Aβ_1–40_ ratio [23]. *CYP46A1* has been associated with AD [24] and correlated with Aβ_1–42_ [25]. *IDE* has been reported to decrease Aβ_1–42_ in AD [26]. Moreover, it has been associated with AD risk [27] and neuropathological Aβ deposition [28].

Thirty-six SNPs in 25 genes were included in the analyses with CSF biomarkers (Table S1). For the genetic association analyses, we tested 25 genetic variants, excluding those in *ABCA7*, *BIN1*, *BDNF, CD2AP*, *CD33, CYP46, CLU*, *CR1, EPHA1, EXOC3L2, MS4A4E* and *MS4A6A* because these genes were previously studied in the Finnish population and found to be associated with AD either in the Finnish cohort alone or in multicenter GWAS [16], [24], [29]–[31].

### Genotyping

DNA was extracted from peripheral blood with EDTA and amplified using polymerase chain reaction technique. DNA samples were randomly placed on 384-well plates. Genotyping using Sequenom iPlex platform (Sequenom, Hamburg, Germany) was performed at University of Eastern Finland (UEF) in Kuopio. Patients and controls were dichotomized as *APOE*-ε4 carriers or noncarriers.

Quality control procedures included using duplicates and negative controls, filtering on individual sample and SNP call rate, and testing whether the SNPs were in Hardy-Weinberg equilibrium (HWE). Samples with an average call rate of 90% were included. SNPs for the association analysis were in Hardy-Weinberg equilibrium (p>0.001).

### CSF Analysis

CSF was obtained through lumbar puncture performed at UEF and the Kuopio University Hospital. Aβ_1–42_, t-tau, and p-tau levels were measured using commercially available INNOTEST enzyme-linked immunosorbent assays (ELISAs) (Innotest ß-amyloid_(1–42)_, Innotest hTau Ag, and Innotest Phospho-tau_(181P)_, Innogenetics, Ghent, Belgium). All measurements were performed at UEF.

The relation between SNPs and biomarkers was assessed only for the AD group because the number of controls with available CSF (n = 30) was too small to allow for meaningful analyses.

### Statistical Analysis

SPSS version 19 (Chicago, IL, USA) was used for the statistical analyses.

We performed power calculations for the genetic association analyses [32]. Simulation analysis yielded more than 80% power to achieve ∼1.3–1.5 risk effect at p = 0.05, indicating that our case-control sample size was sufficient to find moderate genetic association with AD. The same risk effect for the false discovery rate (FDR) corrected p = 0.005 yielded more than 50% power. For quantitative trait association [33], we computed ∼80% power for an effect size of 60–70 pg/ml for the dominant model at p = 0.05 and 50% power at FDR corrected p = 0.005. Similarly for t-tau, the effect size was 120–150 pg/ml and for p-tau 15–20 pg/ml for a power of 80% at p = 0.05 and 50% when FDR corrected (p = 0.005).

Genetic association with AD was examined using Pearson chi-square test for multiple group comparisons and binary logistic regression for pairwise comparisons in univariate and multivariate analysis adjusted for age, sex, and *APOE*-ε4 status. The relation between SNPs and biomarkers was assessed through mixed models for multiple group comparisons and pairwise comparisons, with correction for confounders. Normality was tested and assumed for biomarkers.

For the binary association, we first performed an overall test that provided information on genotype differences between cases and controls, and multiple comparison tests. For the quantitative association, we compared differences in mean biomarker values between genotypes in the overall test. Based on genotype frequencies, we opted to use a dominant model. Minor genotype frequencies for both genetic association and CSF biomarker analyses were 0–15% for 40–50% of the SNPs studied. This reduces statistical power of a recessive model in most SNPs. All analyses were first corrected for age and sex, and then repeated with corrections for age, sex, and *APOE*-ε4 status.

We used FDR correction for multiple testing, following the method of Benjamini and Hochberg [34]. Corrections were based on the number of SNPs tested and were performed separately for binary and quantitative associations. Observed p-values were ranked from smallest to largest. Adjusted p-values were successively computed in a step-up manner, starting from the second largest p-value, as follows: observed p-value (total number of SNPs tested/rank). Statistical significance was set at FDR adjusted p<0.05.

## Results

### Characteristics of the Cohort

Table 1 shows the demographic characteristics of the participants. AD patients with available CSF did not differ from patients without CSF in terms of onset age (p = 0.07) and Mini-Mental State Examination [35] score (p = 0.77).

**Table 1:** Characteristics of study participants.

Characteristics	AD (N = 890)	Control (N = 701)	AD with CSF subgroup (n = 222)
Age, mean (SD), y	69.8 (8.2)^a^	69.1 (6.2)^b^	69 (8)^a^
Female sex (%)	596 (67)	420 (60)	149 (67)
MMSE score, mean (SD)	19 (5)	–	19 (6)
*APOE* ε2/ε3/ε4 allelic distribution, %	2/53/45	4/80/16	–
A**β_1–42_** level (SD), pg/ml		735 (195)^c^	443 (158)^d^
Phosphorylated tau level (SD), pg/ml		63 (26)^e^	84 (36)^f^
Total tau level (SD), pg/ml		311 (143)^g^	546 (269)^h^

Abbreviations: AD, Alzheimer’s disease; CSF, cerebrospinal fluid; N, n, sample size; SD, standard deviation; y, years; MMSE, Mini-Mental State Examination.

a Onset age.

b Age at examination.

c Available for 32 control subjects.

d Available for 222 AD patients.

e Available for 30 control subjects.

f Available for 151 AD patients.

g Available for 30 control subjects.

h Available for 159 AD patients.

### Genetic Association with AD

*APOE* conferred a significant AD risk of 6.30 times higher among *ε*4 allele carriers compared to noncarriers (p<0.0001, Table 2). Age and gender did not affect the results (OR = 6.25, 95% CI 5.22–8.15, p<0.0001).

**Table 2:** SNPs associated with Alzheimer’s disease risk.

Chr, Gene	SNP	Genotypes	Cases (n) genotypic frequencies, %	MAF, %	Controls (n) genotypic frequencies, %	MAF, %	AD risk Overall p-value	Genetic model	Univariate analysis OR(95% CI)	p-value	Multivariate analysis OR(95% CI)	p-value
2, *EPC2*	rs1374441	TT/TC/CC	(868) 58/36/6	24	(681) 59/35/6	24	0.87	TT vs. TC+CC	1.04 (0.85–1.28)	0.69	1.03 (0.82–1.29)	0.83
2, *EPC2*	rs4499362	CC/CT/TT	(875) 57/37/6	25	(689) 58/35/7	24	0.52	CC vs. CT+TT	1.05 (0.86–1.29)	0.61	1.05 (0.84–1.30)	0.69
2, *PPP3R1*	rs1868402	TT/TC/CC	(853) 53/39/8	27	(668) 56/38/6	25	0.30	TT vs. TC+CC	1.13 (0.92–1.38)	0.25	1.29 (1.01–1.62)	**0.03** b
3, *TF*	rs1049296	CC/CT/TT	(866) 81/18/1	10	(680) 81/18/1	10	0.48	CC vs. CT+TT	0.99 (0.77–1.28)	0.93	0.96 (0.72–1.27)	0.76
6, *MTHFD1L*	rs11754661	GG/GA/AA	(878) 92/8/0	4	(690) 92/8/0	4	0.44	GG vs. GA+AA	1.10 (0.76–1.59)	0.61	1.06 (0.71–1.59)	0.77
7, *RELN*	rs4298437	CC/CT/TT	(879) 52/38/10	29	(690) 53/37/10	29	0.98	CC vs. CT+TT	1.02 (0.83–1.24)	0.86	0.90 (0.72–1.13)	0.35
10, *IDE*	rs1887922	TT/TC/CC	(871) 72/26/2	15	(683) 75/23/2	14	0.27	TT vs. TC+CC	1.19 (0.95–1.50)	0.13	1.27 (0.99–1.64)	0.07
11, *MS4A4A*	rs2304933	CC/CA/AA	(882) 35/48/17	41	(690) 34/49/17	41	0.89	CC vs. CA+AA	0.95 (0.77–1.18)	0.66	1.06 (0.84–1.34)	0.61
11, *MS4A4A*	rs4938933	TT/TC/CC	(872) 59/36/5	23	(683) 57/36/7	25	0.12	TT vs. TC+CC	0.93 (0.76–1.14)	0.48	1.02 (0.81–1.27)	0.89
11, *PICALM*	rs3851179	GG/GA/AA	(878) 45/43/12	34	(691) 43/42/15	36	0.32	GG vs. GA+AA	0.93 (0.76–1.14)	0.50	0.97 (0.77–1.21)	0.77
11, *PICALM*	rs642949	TT/TC/CC	(868) 58/36/6	24	(681) 58/37/5	23	0.59	TT vs. TC+CC	1.02 (0.83–1.25)	0.84	1.00 (0.80–1.26)	0.97
11, *SORL1*	rs2070045	TT/TG/GG	(883) 57/37/6	25	(695) 54/39/7	27	0.41	TT vs. TG+GG	0.88 (0.72–1.07)	0.20	0.87 (0.69–1.08)	0.21
11, *SORL1*	rs3824968	TT/TA/AA	(673) 44/39/17	36	(568) 42/43/15	36	0.42	TT vs. TA+AA	0.93 (0.74–1.12)	0.51	0.95 (0.74–1.22)	0.70
11, *SORL1*	rs73595277	CC/CG/GG	(873) 79/20/1	11	(688) 78/20/2	12	0.48	CC vs. CG+GG	0.96 (0.75–1.22)	0.74	0.84 (0.64–1.11)	0.22
15, *CYP19A*	rs2899472	CC/CA/AA	(883) 60/35/5	22	(690) 60/35/5	22	0.98	CC vs. CA+AA	1.01 (0.82–1.23)	0.96	1.02 (0.82–1.28)	0.85
17, *ACE*	rs4293	AA/AG/GG	(866) 30/50/20	45	(682) 26/53/21	47	0.20	AA vs. AG+AA	0.82 (0.66–1.03)	0.08	0.72 (0.56–0.93)	**0.01** a
17, *MAPT*	rs1467967	AA/AG/GG	(869) 43/45/12	34	(685) 42/45/13	35	0.86	AA vs. AG+GG	0.98 (0.78–1.17)	0.67	0.96 (0.77–1.20)	0.72
17, *MAPT*	rs16940758	CC/CT/TT	(886) 65/31/4	20	(700) 63/30/7	22	**0.03** b	CC vs. CT+TT	0.90 (0.73–1.11)	0.33	0.84 (0.67–1.06)	0.13
17, *MAPT*	rs2435211	CC/CT/TT	(880) 37/48/15	39	(691) 37/47/16	38	0.77	CC vs. CT+TT	1.03 (0.84–1.26)	0.80	0.98 (0.78–1.23)	0.84
17, *MAPT*	rs7521	AA/AG/GG	(869) 25/50/25	50	(683) 26/50/24	49	0.83	AA vs. AG+GG	1.05 (0.84–1.32)	0.68	1.02 (0.79–1.31)	0.89
19, *TOMM40*	rs157580	AA/AG/GG	(872) 69/29/2	17	(685) 54/39/7	27	**<0.0001** a	AA vs. AG+GG	0.53 (0.43–0.65)	**<0.0001** a	0.76 (0.60–0.96)	**0.02** b
19, *TOMM40*	rs2075650	AA/AG/GG	(881) 44/44/12	34	(689) 74/23/3	15	**<0.0001** a	AA vs. AG+GG	3.63 (2.92–4.50)	**<0.0001** a	1.46 (1.11–1.92)	**0.007** b
19, *TOMM40*	rs8106922	AA/AG/GG	(886) 43/44/13	35	(695) 26/43/31	53	**<0.0001** a	AA vs. AG+GG	0.46 (0.37–0.57)	**<0.0001** a	1.05 (0.81–1.37)	0.60
19, *APOE*		ε2/ε3/ε4	(890) 29/51/20	–c	(701) 72/24/4	–c	**<0.0001** a	0ε4 vs.1ε4+2ε4	6.30 (5.06–7.85)	**<0.0001** a	6.25 (5.22–8.15)	**<0.0001** a

Abbreviations: SNPs, single nucleotide polymorphisms; Chr, chromosome; n, number of cases; MAF, minor allele frequency; OR, odds ratio; CI, confidence interval; 0ε4, *APOE* ε4 allele noncarriers; 1ε4, carriers of 1 *APOE* ε4 allele; 2ε4, carriers of 2 *APOE* ε4 alleles.

Multiple comparison analysis (overall) was calculated by Pearson chi-square test. Univariate and multivariate analyses were calculated using binary logistic regression assuming a dominant model. Multivariate analyses were adjusted for age, sex and *APOE*-ε4 status. Analyses testing *APOE* adjusted for age and gender. Results for *APOE* are shown for comparative purposes.

a Significant at false discovery rate adjusted *P*<0.05.

b Not significant after false discovery rate correction.

c Minor allele frequency not computed because *APOE* is tri-allelic.

*TOMM40* rs157580, rs2075650, and rs8106922 indicated association with AD risk in the univariate analyses (FDR p<0.05). A protective effect was observed for G allele carriers of rs157580 and rs8106922, and a risk effect for G allele carriers of rs2075650. Only rs2075650 remained significant in the multivariate analysis (unadjusted p<0.007) but did not pass FDR correction (p>0.05).

Results for *MAPT* rs16940758 suggested no association with AD (FDR adjusted p>0.05), although AD and control groups differed in multiple comparison test. *PPP3R1* rs1868402 and *ACE* rs4293 showed association with AD in multivariate analysis (unadjusted p = 0.03 for rs1868402 and p = 0.01 for rs4293) but did not remain significant after FDR adjustment. Overall and univariate analyses with rs1868402 and rs4293 were not significant.

### Effects of SNPS on CSF Aβ_1–42_

*APOE* ε4 allele carriers had significantly reduced CSF Aβ_1–42_ (FDR adjusted p<0.05, Table 3). Apart from *APOE,* only *CLU* significantly affected Aβ_1–42_ among the AlzGene top loci. Carriers of rs11136000 major allele (C, risk allele in AlzGene meta-analysis) showed significantly decreased Aβ_1–42_ (FDR adjusted p<0.05).

**Table 3:** Effects of SNPs on CSF Aβ_1–42_ in Alzheimer’s disease.

Chr, Gene	SNP	Genotypes	Genotype, n	Mean (SD) Aβ_1–42_ level, pg/ml, per genotype	p-value	
					Overall	DM
Top AlzGene loci						
1, *CR1*	rs6656401	GG/GA/A**A**	127/82/8	446(168)/443(142)/370(150)	0.48 (0.21)	0.82 (0.96)
2, *BIN1*	rs744373	TT/TC/C**C**	117/96/8	446(148)/440(161)/451(266)	0.97 (0.92)	0.94 (1.00)
2, *BIN1*	rs7561528	GG/GA/AA	99/97/25	447(152)/441(163)/443(173)	0.92 (0.71)	0.85 (0.77)
6, *CD2AP*	rs9349407	GG/GC/C**C**	132/73/15	440(158)/450(152)/444(202)	0.97 (0.88)	0.84 (0.84)
8, *CLU*	rs11136000	**C**C/CT/TT	75/112/32	400(116)/459(166)/497(194)	0.005 (0.01)	**0.003** a **(0.005)**
11, *MS4A4E*	rs670139	AA/AC/C**C**	83/111/27	465(164)/436(155)/408(152)	0.18 (0.18)	0.13 (0.11)
11, *MS4A6A*	rs610932	**C**C/CA/AA	116/87/17	447(153)/443(163)/410(176)	0.67 (0.74)	0.73 (0.51)
11, *PICALM*	rs642949	TT/TC/CC	130/79/11	459(165)/432(148)/346(116)	0.07 (0.06)	0.12 (0.11)
11, *PICALM*	rs3851179	**G**G/GA/AA	96/104/21	441(172)/453(149)/412(140)	0.52 (0.34)	0.95 (0.61)
19, *ABCA7*	rs3752246^§^	CC/CG/GG	165/40/4	445(160)/446(159)/433(281)	0.99 (0.83)	0.90 (0.91)
19, *CD33*	rs3865444	**G**G/GT/TT	89/111/21	437(169)/448(155)/441(135)	0.87 (0.78)	0.65 (0.48)
19, *APOE*		ε2/ε3/**ε4**	50/111/61	532 (214)/441(136)/374 (95)	<0.001	**<0.001** a
Selection GWAS SNPs not in AlzGene top						
6, *MTHFD1L*	rs11754661	GG/GA/AA	206/15/0	440(156)/490(189)/−c	0.20 (0.07)	0.20 (0.07)
11, *MS4A4A*	rs2304933	CC/CA/A**A**	72/107/43	494(192)/428(133)/395(130)	0.002 (0.001)	0.001^a^ (<0.0001)
11, *MS4A4A*	rs4938933	TT/TC/CC	125/81/14	453(160)/435(152)/411(188)	0.60 (0.54)	0.41 (0.29)
19, *EXOC3L2*	rs597668	TT/TC/C**C**	97/98/24	472(192)/422(123)/423(129)	0.08 (0.56)	0.02 (0.28)
GWAS with biomarkers as outcome						
2, *EPC2*	rs1374441	TT/TC/CC	119/85/15	440(172)/456(147)/411(109)	0.52 (0.38)	0.67 (0.58)
2, *EPC2*	rs4499362	CC/CT/TT	119/89/13	443(172)/445(140)/441(155)	0.98 (1.00)	0.96 (0.98)
15, *CYP19A*	rs2899472	CC/CA/AA	132/79/11	454(163)/433(152)/378(122)	0.36 (0.22)	0.27 (0.19)
7, *RELN*	rs429837	CC/CT/TT	111/83/27	439(137)/432(156)/498(230)	0.17 (0.21)	0.86 (0.79)
19, *TOMM40*	rs157580	**A**A/AG/GG	150/68/3	426(146)/476(175)/593(202)	0.04 (0.27)	**0.02** b **(0.12)**
19, *TOMM40*	rs2075650	AA/AG/G**G**	98/100/24	485(195)/418(112)/377(104)	0.002 (0.24)	**0.001** a **(0.19)**
Other candidate genes						
2, *PPP3R1*	rs1868402	TT/TC/CC	121/75/18	447(173)/448(145)/418(131)	0.70 (0.56)	0.81 (0.53)
3, *TF*	rs1049296	CC/CT/T**T**	176/40/4	449(165)/412(120)/539(146)	0.22 (0.37)	0.42 (0.42)
10, *IDE*	rs1887922	TT/TC/C**C**	167/52/2	442(138)/437(196)/747(457)	0.03 (0.04)	0.82 (0.94)
11, *BDNF*	rs6265	GG/GA/A**A**	128/45/3	430(156)/453(173)/432(142)	0.79 (0.62)	0.49 (0.35)
11, *SORL1*	rs2070045	TT/TG/G**G**	122/86/13	440(149)/453(166)/403(195)	0.58 (0.53)	0.89 (0.89)
11, *SORL1*	rs3824968	TT/TA/A**A**	58/66/35	438(130)/442(158)/457(206)	0.92 (0.87)	0.85 (0.68)
11, *SORL1*	rs73595277	CC/CG/GG	183/36/2	449(168)/421(102)/397(76)	0.65 (0.87)	0.35 (0.60)
14, *CYP46a*	rs754203	TT/TC/CC	103/98/20	447(155)/467(160)/456(176)	0.85 (0.66)	0.72 (0.54)
17, *ACE*	rs4293	**A**A/AG/GG	60/107/54	451(184)/451(154)/421(136)	0.26 (0.67)	0.56 (0.91)
17, *MAPT*	rs16940758	CC/CT/TT	146/68/8	439(169)/449(141)/467(99)	0.90 (0.84)	0.68 (0.57)
17, *MAPT*	rs2435211	CC/CT/TT	71/118/32	420(136)/456(167)/450(171)	0.29 (0.27)	0.12 (0.17)
17, *MAPT*	rs1467967	**A**A/AG/GG	97/98/25	446(168)/451(161)/409(100)	0.58 (0.57)	0.81 (0.99)
17, *MAPT*	rs7521	AA/AG/G**G**	62/112/46	419(105)/456(179)/449(165)	0.37 (0.33)	0.17 (0.14)
19, *TOMM40*	rs8106922	AA/AG/GG	113/91/18	417(126)/461(169)/520(236)	0.02 (0.75)	**0.02** b **(0.55)**

Abbreviations: Chr, chromosome; SNP, single nucleotide polymorphism; n, number of cases; SD, standard deviation; DM, dominant model; GWAS, genome-wide association study.

Risk allele according to AlzGene meta-analyses or study source in **bold** and underscored. Information on risk allele was not available for all studies. *P*-values based on mixed model.

analyses adjusted for age and gender; values in parenthesis () adjusted for age, gender and *APOE*-ε4 status. Analyses testing *APOE* adjusted for age and gender. Results for *APOE* are shown for comparative purposes.

a Significant at false discovery rate corrected *P*<0.05.

b No longer significant after false discovery rate correction.

c No cerebrospinal fluid measured because none of the participants carried the rs11754661 AA genotype.

Of the SNPs identified in GWAS but not in the top AlzGene loci, only *MS4A4A* correlated with Aβ_1–42._ Minor allele carriers (A, risk allele in GWAS) of rs2304933 showed significantly decreased Aβ_1–42_ levels (FDR adjusted p<0.05) compared to major allele (C) carriers. The correlation was strengthened when corrected for *APOE*-ε4 status.

Decreased Aβ_1–42_ levels among *EXOC3L2* rs597668 minor allele carriers (C, risk allele in GWAS) were observed (unadjusted p = 0.02) but the correlation did not pass FDR filter (p>0.05).

Risk allele carriers of *TOMM40* SNPs identified through GWAS with biomarkers as outcome and through other candidate gene studies had decreased Aβ_1–42_ concentrations, but this was not independent of *APOE*-ε4 status (FDR adjusted p>0.05).

The rest of the SNPs did not exhibit conclusive effects on Aβ_1–42_ concentrations.

### Effects of SNPs on CSF Tau

None of the SNPs from the top AlzGene loci significantly correlated with CSF t-tau and p-tau. The strongest effect was for *PICALM* rs642949 and t-tau (p = 0.06, adjusted for age, sex, and *APOE*-ε4 status), with increased t-tau levels among minor allele (C) carriers (Table 4). *APOE* ε4 allele carriers also showed a nonsignificant increase in t-tau concentrations (p = 0.08).

**Table 4:** Effects of SNPs on CSF t-tau and p-tau in Alzheimer’s disease.

Chr, Gene	SNP	Genotypes	Genotype, n	Mean (SD) t-tau level, pg/ml, per genotype	p-value		Genotype, n	Mean (SD) p-tau level, pg/ml, per genotype	p-value	
					Overall	DM			Overall	DM
Top AlzGene loci										
1, *CR1*	rs6656401	GG/GA/A**A**	93/57/7	512(246)/579(275)/682(456)	0.48 (0.44)	0.28 (0.30)	90/53/7	81(33)/88(35)/103(66)	0.53 (0.48)	0.40 (0.41)
2, *BIN1*	rs744373	TT/TC/C**C**	80/74/5	576(287)/514(251)/547(224)	0.29 (0.26)	0.11 (0.10)	76/69/6	86(36)/81(35)/100(44)	0.41 (0.41)	0.47 (0.45)
2, *BIN1*	rs7561528	GG/GA/AA	68/72/19	583(281)/511(247)/544(305)	0.26 (0.26)	0.10 (0.10)	65/66/20	86(34)/82(37)/86(38)	0.87 (0.87)	0.63 (0.62)
6, *CD2AP*	rs9349407	GG/GC/C**C**	94/54/10	551(281)/537(245)/566(305)	0.95 (0.89)	0.95 (0.84)	90/50/10	86(39)/82(31)/87(41)	0.87 (0.83)	0.74 (0.69)
8, *CLU*	rs11136000	**C**C/CT/TT	59/79/19	523(230)/543(274)/564(299)	0.77 (0.65)	0.47 (0.36)	59/72/18	83(36)/83(35)/88(40)	0.88 (0.85)	0.80 (0.72)
11, *MS4A4E*	rs670139	AA/AC/C**C**	60/81/18	556(286)/519(233)/634(351)	0.20 (0.25)	0.68 (0.78)	55/77/19	83(37)/83(35)/93(37)	0.49 (0.50)	0.70 (0.66)
11, *MS4A6A*	rs610932	**C**C/CA/AA	77/71/9	572(281)/525(250)/537(337)	0.57 (0.67)	0.30 (0.42)	74/66/9	84(35)/83(33)/96(62)	0.73 (0.76)	0.83 (0.75)
11, *PICALM*	rs3851179	**G**G/GA/AA	75/69/15	583(301)/518(243)/488(194)	0.49 (0.40)	0.25 (0.20)	72/65/14	86(39)/84(36)/76(20)	0.80 (0.76)	0.76 (0.72)
11, *PICALM*	rs642949	TT/TC/CC	94/56/8	499(221)/612(313)/525(223)	0.11 (0.10)	0.07 (0.06)	89/54/7	79(32)/93(39)/75(37)	0.13 (0.14)	0.15 (0.15)
19, *ABCA7*	rs3752246	CC/CG/GG	119/25/4	532(267)/570(275)/568(185)	0.98 (0.98)	0.83 (0.89)	111/26/4	81(36)/93(37)/82(30)	0.43 (0.45)	0.25 (0.56)
19, *CD33*	rs3865444	**G**G/GT/TT	63/81/14	587(305)/503(202)/622(394)	0.11 (0.12)	0.12 (0.10)	61/75/14	87(45)/82(26)/87(43)	0.62 (0.60)	0.37 (0.34)
19, *APOE*		ε2/ε3/**ε4**	37/79/43	468 (289)/537(249)/630 (269)	0.11	0.08	36/75/40	78(42)/84(35)/91(31)	0.54	0.36
Selection GWAS SNPs not in AlzGene top										
6, *MTHFD1L*	rs11754661	GG/GA/AA	149/10/0	550(268)/492(301)/a	0.48 (0.39)	0.48 (0.39)	141/10/0	85(36)/72(38)/a	0.26 (0.22)	0.26 (0.22)
11, *MS4A4A*	rs2304933	CC/CA/A**A**	54/76/29	526(293)/534(233)/611(309)	0.46 (0.39)	0.60 (0.56)	50/71/30	81(40)/85(35)/88(33)	0.18 (0.79)	0.56 (0.55)
11, *MS4A4A*	rs4938933	TT/TC/CC	86/64/8	561(270)/509(238)/567(347)	0.41 (0.51)	0.18 (0.24)	82/60/8	86(37)/82(33)/80(40)	0.59 (0.61)	0.36 (0.39)
19, *EXOC3L2*	rs597668	TT/TC/C**C**	73/70/14	538(270)/562(271)/446(136)	0.30 (0.18)	0.73 (0.32)	70/64/15	85(41)/84(33)/78(20)	0.72 (0.58)	0.54 (0.37)
GWAS with biomarkers as outcome										
2, *EPC2*	rs1374441	TT/TC/C**C**	87/60/10	511(239)/567(269)/631(387)	0.14 (0.13)	0.06 (0.06)	85/55/9	80(30)/86(38)/102(63)	0.17 (0.16)	0.13 (0.13)
2, *EPC2*	rs4499362	CC/CT/TT	88/62/9	539(293)/559(242)/528(221)	0.68 (0.61)	0.41 (0.35)	86/58/7	84(37)/86(36)/73(26)	0.53 (0.48)	0.61 (0.57)
15, *CYP19A*	rs2899472	CC/CA/AA	98/51/10	534(274)/567(235)/557(387)	0.76 (0.77)	0.77 (0.79)	90/51/10	84(38)/86(31)/80(44)	0.69 (0.68)	0.84 (0.84)
7, *RELN*	rs429837	CC/CT/TT	73/63/23	519(255)/588(264)/516(318)	0.17 (0.23)	0.13 (0.15)	68/62/21	83(35)/90(37)/73(35)	0.13 (0.16)	0.53 (0.60)
19, *TOMM40*	rs157580	**A**A/AG/GG	113/43/2	567(281)/492(226)/329(250)	0.27 (0.42)	0.15 (0.21)	107/41/2	85(36)/83(38)/15(53)	0.56 (0.66)	0.86 (0.96)
19, *TOMM40*	rs2075650	AA/AG/G**G**	74/71/14	505(274)/573(251)/623(315)	0.52 (0.96)	0.26 (0.81)	72/66/13	81(36)/87(36)/87(32)	0.80 (0.92)	0.59 (0.91)
Other candidate genes										
2, *PPP3R1*	rs1868402	TT/TC/CC	95/50/9	515(173)/624(316)/497(264)	0.07 (0.04)	0.05 (0.03)^b^	91/46/9	82(33)/93(41)/78(40)	0.24 (0.20)	0.20 (0.17)
3, *TF*	rs1049296	CC/CT/T**T**	128/27/3	547(285)/543(189)/563(310)	0.83 (0.84)	0.55 (0.56)	120/27/3	82(35)/94(41)/80(43)	0.53 (0.51)	0.35 (0.32)
10, *IDE*	rs1887922	TT/TC/C**C**	117/40/2	547(275)/551(259)/368(145)	0.63 (0.68)	0.97 (0.92)	112/37/2	84(35)/87(39)/65(34)	0.69 (0.71)	0.80 (0.76)
11, *BDNF*	rs6265	GG/GA/A**A**	98/30/2	543(266)/492(182)/777(350)	0.50 (0.50)	0.45 (0.41)	92/29/2	84(37)/79(31)/121(57)	0.45 (0.45)	0.75 (0.74)
11, *SORL1*	rs2070045	TT/TG/G**G**	86/63/9	583(288)/503(236)/532(270)	0.41 (0.43)	0.18 (0.20)	83/58/9	86(36)/81(36)/89(36)	0.84 (0.85)	0.79 (0.80)
11, *SORL1*	rs3824968	TT/TA/A**A**	38/41/28	530(284)/522(245)/557(223)	0.55 (0.57)	0.50 (0.55)	36/41/24	87(43)/83(36)/89(29)	0.72 (0.72)	0.96 (0.99)
11, *SORL1*	rs73595277	CC/CG/GG	132/25/2	529(241)/624(388)/698(16)	0.18 (0.23)	0.07 (0.09)	127/22/2	82(32)/98(53)/91(40)	0.11 (0.12)	0.04^b^ (0.05)
14, *CYP46a*	rs754203	TT/TC/CC	73/74/11	564(281)/526(257)/479(116)	0.64 (0.73)	0.37 (0.46)	69/71/10	81(34)/88(39)/73(17)	0.36 (0.32)	0.31 (0.26)
17, *ACE*	rs4293	**A**A/AG/GG	43/82/34	601(262)/506(263)/574(284)	0.08 (0.09)	0.31 (020)	41/78/32	86(29)/83(41)/84(33)	0.84 (0.89)	0.96 (0.94)
17, *MAPT*	rs16940758	CC/CT/TT	108/46/5	526(264)/579(282)/657(258)	0.11 (0.10)	0.06 (0.05)	105/41/5	80(36)/93(37)/90(24)	0.08 (0.08)	0.03^b^ (0.02)^b^
17, *MAPT*	rs2435211	CC/CT/TT	50/86/23	483(184)/562(34)/621(370)	0.08 (0.05)	0.03^b^ (0.02)^b^	47/81/23	80(32)/84(34)/95(49)	0.27 (0.22)	0.22 (0.20)
17, *MAPT*	rs1467967	AA/AG/G**G**	64/74/20	577(303)/515(251)/552(215)	0.59 (0.50)	0.37 (0.28)	61/70/19	89(40)/80(35)/84(26)	0.62 (0.59)	0.36 (0.32)
17, *MAPT*	rs7521	AA/AG/GG	42/84/32	543(240)/534(266)/554(281)	0.91 (0.92)	0.78 (0.71)	41/76/33	85(30)/78(32)/95(46)	0.07 (0.07)	0.98 (0.92)
19, *TOMM40*	rs8106922	**A**A/AG/GG	78/66/15	570(257)/526(257)/512(375)	0.75 (0.82)	0.49 (0.93)	36/75/40	87(35)/82(33)/82(53)	0.88 (0.94)	0.63 (0.90)

Abbreviations: Chr, chromosome; SNP, single nucleotide polymorphism; n, number of cases; SD, standard deviation; t-tau, total tau; p-tau, phosphorylated tau; DM, dominant model; GWAS, genome-wide association studies.

Risk allele according to AlzGene meta-analyses or study source in **bold** and underscored. Information on risk allele was not available for all studies. All CSF values are means (standard deviation). *P*-values based on mixed model analyses adjusted for age and gender; values in parenthesis () adjusted for age, gender and *APOE*-ε4 status. Analyses testing *APOE* adjusted for age and gender. Results for *APOE* are shown for comparative purposes.

a No cerebrospinal fluid measured because none of the participants carried the rs11754661 AA genotype.

b No longer significant after false discovery rate correction.

None of the polymorphisms in other GWAS with clinical AD as outcome were linked with t-tau and p-tau.

From GWAS SNPs with biomarker as outcome, none of the results attained statistical significance. *EPC2* rs1374441 and t-tau showed the strongest effect. T-tau levels increased among minor allele (C, risk allele in GWAS) carriers (p = 0.06, corrected for age, sex, and *APOE*-ε4 status).

Of the SNPs selected from other candidate genes, marginal correlations at FDR unadjusted p<0.05 were obtained for polymorphisms in *PPP3R1, SORL1,* and *MAPT* (Table 4). T-tau levels increased among carriers of *PPP3R1* rs1868402 major allele (T, FDR adjusted p>0.05). The effect was strengthened when corrected for *APOE*-ε4 status.

*SORL1* rs73595277 minor allele carriers (G) had increased p-tau (FDR adjusted p>0.05). Effects of *SORL1* slightly decreased with *APOE*-ε4 correction.

In *MAPT*, minor allele carriers of rs2435211 (T) had increased t-tau levels (unadjusted p = 0.03) and minor allele carriers of rs16940758 had increased p-tau levels (unadjusted p = 0.03). Correcting for *APOE*-ε4 status enhanced the effects. These results, however, did not remain significant after FDR correction (adjusted p>0.05 for both SNPs).

## Discussion

We performed a case-control genetic association analysis of 25 AD risk variants and tested the effects of 36 risk variants on CSF Aβ_1–42,_ t-tau, and p-tau in the AD group.

The allele frequencies of genes from AlzGene meta-analysis of Caucasian ancestry were comparable with allele frequencies of most SNPs in our study. Only *APOE* and *TOMM40* showed genetic association with AD. However, *TOMM40* is in linkage disequilibrium with *APOE* and did not exhibit an effect independent of *APOE*, in accordance with recent evidence [36].

Of the AlzGene top loci, *APOE* and *CLU* correlated significantly with decreased Aβ_1–42._ Our result for *APOE* confirms previous findings [37]. Other studies on *CLU* have not found the same effect, which may be attributed to their smaller sample size [38], [39]. Consistent with GWAS findings, the minor allele of *CLU* exerted a protective effect on AD risk [29], and on CSF Aβ_1–42_ in the Finnish cohort. *CLU* did not significantly affect tau_._ *CLU* binds soluble Aβ and plays a role in Aβ clearance and aggregation [14], which could partly explain why it primarily affected Aβ.

*PICALM* affects Aβ concentration in the brain through endocytic processes. Rs3851179 has been reported to correlate with CSF Aβ_1–42_ in another study, the major allele (G) being the risk allele [39]. In our cohort, we found no correlation between rs3851179 and CSF markers. Instead, rs642949 minor allele carriers (C) had decreased Aβ_1–42_ and increased t-tau levels, although these were not statistically significant. The C allele of rs642949 has been reported to exert a risk effect in a case-control study [40].

*APOE* and the other top AlzGene loci did not correlate with t-tau and p-tau in our cohort.

Of the GWAS SNPs not in AlzGene top loci, *MS4A4A* showed significant correlation with Aβ_1–42_ but not with tau. This is a novel finding. *MS4A4A* belongs to the *MS4A* cluster [18]. Not much is known yet about *MS4A4A* rs2304933 but its effect on Aβ_1–42_ is consistent with our genetic association analysis results suggesting a risk effect of the minor (A) allele. The mechanisms by which *MS4A4A* affect CSF Aβ_1–42_ levels need further investigation.

The decrease in Aβ_1–42_ levels among C allele carriers of *EXOC3L2* rs597668 is interesting. Although not statistically significant (FDR adjusted p>0.05), it conferred with a previous study identifying the C allele as a risk allele in the Finnish population [30]. The C allele has also been reported in another study to promote AD progression [41].

Among GWAS SNPs with biomarker as outcome, *TOMM40* SNPs correlated with CSF Aβ_1–42_ but not with tau, which was consistent with previous findings [19], [20]. None of the *TOMM40* SNPs remained significant after *APOE* correction.

The SNPs from candidate genes were not related to CSF Aβ_1–42_. For tau, the small effects we observed for variants in *PPP3R1, SORL1,* and *MAPT* (unadjusted p<0.05) were suggestive of a trend. *PPP3R1* is a protein phosphatase and is the calcium binding regulatory subunit of calcineurin [42]. Calcineurin is involved in modulating tau phosphorylation [43]. A previous study found *PPP3R1* to correlate with p-tau [4]. In our cohort, we found an effect on t-tau but the effect on p-tau was weaker. This difference in results could partly be attributed to variability in population.

*SORL1* binds to ApoE and plays a role in Aβ_1–42_ production [37]. Numerous SNPs in *SORL1* have been studied in relation to AD [44] and CSF biomarkers [37] but results so far have been inconclusive [9]. One study found that rs3824968 in *SORL1* significantly reduced Aβ_1–42_ in AD [45] whereas another study reported no correlation [46]. *SORL1* appears to exert small effects likely to be detected only in mega-analysis of pooled samples [47] or in haplotypes [48]. In general, single loci in *SORL1* did not correlate with CSF Aβ_1–42_ and tau [9]. Our result relating *SORL* with p-tau may be due to chance.

*MAPT* codes for tau proteins. Aggregated hyperphosphorylated tau proteins are a component of NFTs. Consistent with the findings of another study, we found *MAPT* to correlate with CSF t-tau [49].

We noted a number of changes in the strength of correlation when we corrected for *APOE*. *TOMM40* was no longer associated with AD and CSF Aβ_1–42_. This confirms previous finding that *TOMM40* is in strong linkage disequilibrium with *APOE.* The effect of *EXOC3L2* on CSF Aβ_1–42_ became nonsignificant. This suggests that the effect was not independent of *APOE*. Due to our small sample size, our result could also be a false positive. For the other SNPs, we found no or minor changes on either AD risk or correlation with CSF markers after *APOE* correction. This means that the effects of these SNPs were independent of *APOE*, or that our sample size was too small to detect interaction effects.

We found few SNP-related differences in CSF Aβ because Aβ may already be strongly decreased in AD patients who were already demented. The effect of genetic risk factors on amyloid metabolism may be more evident in predementia stages. Of interest is the weak correlation of *MAPT* and *PPP3R1* with tau, which confirms the role of these SNPs in tau metabolism. *MAPT* and *PPP3R1* also showed weak associations with clinical AD, which suggest that they contribute to dementia risk. In the analyses with biomarkers, none of the AlzGene or other GWAS SNPs were related to tau, suggesting that these SNPs have no clear effect on tau metabolism.

Our study had the unique design that we tested SNPs both for genetic association in a clinical case-control design and for correlation with CSF biomarkers. Another strength was the large selection of high-risk SNPs identified by GWAS or other candidate gene studies covering different possible pathophysiological pathways. The small sample size remained a limitation. We had sufficient power for finding moderate associations, but a larger sample is needed for detecting the very small effect sizes of the other SNPs studied, if these effects are present.

In conclusion, we provide evidence that *APOE*, *CLU,* and *MS4A4A,* which have been identified in GWAS to be associated with AD, also significantly affected CSF Aβ_1–42._ To our knowledge, ours is the first study to report on the correlation between *MS4A4A* and CSF Aβ_1–42._ None of the AD risk genes studied showed significant effects on CSF tau. The nonsignificant trends in *PPP3R1* and *MAPT* in relation to tau may be due to our small sample size rather than genuine lack of risk effects. Collaboration on a larger scale is necessary to ascertain the effects of the aforementioned SNPs and identify reliable genetic risk variants for AD markers in CSF.

## Supporting Information

Table S1 **Genetic variants included in the study.** We listed here the 36 SNPs that were all tested for correlation with CSF Aβ_1–42_ and tau. We indicated which SNPs were excluded from the genetic association analysis because they have been previously genotyped and reported in genetic association studies including the Finnish cohort.
(DOC)
