# Supplementary material for: Genetic Loci Associated with Alzheimer’s Disease and Cerebrospinal Fluid Biomarkers in a Finnish Case-Control Cohort
Source: PLoS One. 2013 Apr 3;8(4):e59676. doi: 10.1371/journal.pone.0059676 (PMC3616106; doi:10.1371/journal.pone.0059676)
Supplement: Table S1 — Genetic variants included in the study. We listed here the 36 SNPs that were all tested for correlation with CSF Aβ1–42 and tau. We indicated which SNPs were excluded from the genetic association analysis because they have been previously genotyped and reported in genetic association studies including the Finnish cohort. (DOC) [file pone.0059676.s001.doc]

**Supporting information for the study:**

Genetic loci associated with Alzheimer’s disease and cerebrospinal fluid biomarkers

in a Finnish case-control cohort

Lyzel S Elias-Sonnenschein, Seppo Helisalmi, Teemu Natunen, Anette Hall, Teemu Paajanen, Sanna-Kaisa Herukka, Marjo Laitinen, Anne M Remes, Anne M Koivisto, Kari M Mattila, Terho Lehtimäki, Frans RJ Verhey, Pieter Jelle Visser, Hilkka Soininen, Mikko Hiltunen

**Table S1. Genetic variants included in the study. All were tested for biomarker analyses. SNPs tested in previous studies for genetic association with AD were not included in the present study, as indicated.**

| **Gene** | **SNP** | **Genetic association** |
| --- | --- | --- |
| ***Top AlzGene loci*** |  |  |
| 1, *CR1* | rs6656401 | Previously tested. Significant in GWAS^a^ |
| 2, *BIN1* | rs744373 | Previously tested. Significant in replication study^a^ |
| 2, *BIN1* | rs7561528 | Gene previously tested in GWAS^a^ |
| 6, *CD2AP* | rs9349407 | Previously tested. Significant in GWAS^a^ |
| 8, *CLU* | rs11136000 | Previously tested. Significant in GWAS^a^ |
| 11, *MS4A4E* | rs670139 | Previously tested. Significant in GWAS^a^ |
| 11, *MS4A6A* | rs610932 | Previously tested. Significant in GWAS^a^ |
| 11, *PICALM* | rs3851179 | Included in current study |
| 11, *PICALM* | rs642949 | Included in current study |
| 19, *ABCA7* | rs3752246 | Previously tested. Significant in GWAS^a^ |
| 19, *CD33* | rs3865444 | Previously tested. Significant in GWAS^a^ |
| 19, *APOE*^c^ | ε2/ε3/ε4 | Included in current study |
| ***GWAS*** |  |  |
| 6, *MTHFD1L* | rs11754661 | Included in current study |
| 11, *MS4A4A* | rs2304933 | Included in current study |
| 11, *MS4A4A* | rs4938933 | Included in current study |
| 19*, EXOC3L2* | rs597668 | Previously tested. Significant in replication study^a^ |
| 2. *EPC2* | rs1374441 | Included in current study |
| 2. *EPC2* | rs4499362 | Included in current study |
| 15, *CYP19A* | rs2899472 | Included in current study^b^ |
| 7, *RELN* | rs429837 | Included in current study |
| 19, *TOMM40* | rs157580 | Included in current study |
| 19, *TOMM40* | rs2075650 | Included in current study |
| ***Other candidate genes*** |  |  |
| 2, *PPP3R1* | rs1868402 | Included in current study |
| 3, *TF* | rs1049296 | Included in current study |
| 10, *IDE* | rs1887922 | Included in current study^b^ |
| 11, *BDNF* | rs6265 | Previously tested. Not significant in genetic association analysis^a^ |
| 11, *SORL1* | rs2070045 | Included in current study |
| 11, *SORL1* | rs3824968 | Included in current study |
| 11, *SORL1* | rs73595277 | Included in current study |
| 14, *CYP46a* | rs754203 | Previously tested. Significant in candidate gene analysis^a^ |
| 17, *ACE* | rs4293 | Included in current study^b^ |
| 17, *MAPT* | rs16940758 | Included in current study |
| 17, *MAPT* | rs2435211 | Included in current study |
| 17, *MAPT haplotype* | rs1467967 | Included in current study |
| 17, *MAPT haplotype* | rs7521 | Included in current study |
| 19, *TOMM40* | rs8106922 | Included in current study |

Abbreviations: Chr, chromosome; SNP, single nucleotide polymorphism; CSF, cerebrospinal fluid; GWAS, genome-wide association study

**Gene names**: *CR1*, complement component receptor 1; *BIN1,* bridging integrator 1; *CD2AP,* CD2-associated protein; *CLU*, clusterin; *MS4A4E,* membrane-spanning 4-domains, subfamily A, member 4E; *MS4A6A*, membrane-spanning 4-domains, subfamily A, member 6A; *PICALM*, phosphatidylinositol binding clathrin assembly protein; *ABCA7,* ATP-binding cassette, subfamily A, member 7; *CD33*, CD33 molecule; *MTHFD1L*, methylenetetrahydrofolate dehydrogenase 1-like; *RELN*, reelin; *MS4A4A*, membrane-spanning 4-domains, subfamily A, member 4A; *EXOC3L2*, exocyst complex component 3-like 2; *EPC2*, enhancer of polycomb homolog 2; *CYP19A*, cytochrome P450, family 19, subfamily a, polypeptide 1; *TOMM40*, translocase of outer mitochondrial membrane 40 homolog; *PPP3R1*, protein phosphatase 3, regulatory subunit B, alpha; *TF*, transferin ; *IDE,* insulin-degrading enzyme; *BDNF*, brain-derived neurotrophic factor; *SORL1*, sortilin-related receptor; *CYP46a*, cytochrome P450, family 46, subfamily A, polypeptide; *ACE*, angiotensin I converting enzyme 1; *MAPT*, microtubule-associated protein tau; *TOMM40*, translocase of outer mitochondrial membrane 40 homolog; *APOE*, apolipoprotein E

^a^Gene previously genotyped in GWAS or SNP genotyped in genetic association studies that included the Finnish cohort and excluded from the genetic association analysis in the present study but included in the biomarker analysis

^b^Gene previously genotyped in the Finnish cohort but with different SNP(s). Present SNP included in the genetic association and biomarker analysis.

^c^*APOE* tested for reference purposes.

Number in table text [ ] denotes references.

**References**

1. Lambert JC, Heath S, Even G, Campion D, Sleegers K, et al. (2009) Genome-wide association study identifies variants at CLU and CR1 associated with Alzheimer's disease. Nat Genet 41: 1094-1099.

2. Lambert JC, Zelenika D, Hiltunen M, Chouraki V, Combarros O, et al. (2011) Evidence of the association of BIN1 and PICALM with the AD risk in contrasting European populations. Neurobiol Aging 32: 756 e711-755.

3. Hollingworth P, Harold D, Sims R, Gerrish A, Lambert JC, et al. (2011) Common variants at ABCA7, MS4A6A/MS4A4E, EPHA1, CD33 and CD2AP are associated with Alzheimer's disease. Nat Genet 43: 429-435.

4. Iivonen S, Corder E, Lehtovirta M, Helisalmi S, Mannermaa A, et al. (2004) Polymorphisms in the CYP19 gene confer increased risk for Alzheimer disease. Neurology 62: 1170-1176.

5. Vepsalainen S, Parkinson M, Helisalmi S, Mannermaa A, Soininen H, et al. (2007) Insulin-degrading enzyme is genetically associated with Alzheimer's disease in the Finnish population. J Med Genet 44: 606-608.

6. Vepsalainen S, Castren E, Helisalmi S, Iivonen S, Mannermaa A, et al. (2005) Genetic analysis of BDNF and TrkB gene polymorphisms in Alzheimer's disease. J Neurol 252: 423-428.

7. Helisalmi S, Vepsalainen S, Koivisto AM, Mannermaa A, Iivonen S, et al. (2006) Association of CYP46 intron 2 polymorphism in Finnish Alzheimer's disease samples and a global scale summary. J Neurol Neurosurg Psychiatry 77: 421-422.

8. Sarajarvi T, Helisalmi S, Antikainen L, Makinen P, Koivisto AM, et al. (2010) An association study of 21 potential Alzheimer's disease risk genes in a Finnish population. J Alzheimers Dis 21: 763-767.
